# Sinomenine Suppresses Development of Hepatocellular Carcinoma Cells *via* Inhibiting MARCH1 and AMPK/STAT3 Signaling Pathway

**DOI:** 10.3389/fmolb.2021.684262

**Published:** 2021-06-10

**Authors:** Wei Yang, Qihua Feng, Minjing Li, Jiaqi Su, Peiyuan Wang, Xu Wang, Yancun Yin, Xia Wang, Mingdong Zhao

**Affiliations:** ^1^ Department of Medical Imaging, Binzhou Medical University, Yantai, China; ^2^ Department of Imaging, Yantai Affiliated Hospital of Binzhou Medical University, Yantai, China; ^3^ Department of Chinese Medicine Prescription, Binzhou Medical University, Yantai, China; ^4^ Department of Human Anatomy, Binzhou Medical University, Yantai, China; ^5^ Department of Oral Pathology, Binzhou Medical University, Yantai, China

**Keywords:** hepatocellular carcinoma, sinomenine, proliferation, AMPK, STAT3, MARCH1

## Abstract

Promotion of apoptosis and suppression of proliferation in tumor cells are popular strategies for developing anticancer drugs. Sinomenine (SIN), a plant-derived alkaloid, displays antitumor activity. However, the mechanism of action of SIN against hepatocellular carcinoma (HCC) is unclear. Herein, several molecular technologies, such as Western Blotting, qRT-PCR, flow cytometry, and gene knockdown were applied to explore the role and mechanism of action of SIN in the treatment of HCC. It was found that SIN arrests HCC cell cycle at G0/G1 phase, induces apoptosis, and suppresses proliferation of HCC cells *via* down-regulating the expression of membrane-associated RING-CH finger protein 1 (MARCH1). Moreover, SIN induces cell death and growth inhibition through AMPK/STAT3 signaling pathway. MARCH1 expression was silenced by siRNA to explore its involvement in the regulation of AMPK/STAT3 signaling pathway. Silencing MARCH1 caused down-regulation of phosphorylation of AMPK, STAT3 and decreased cell *via*bility and function. Our results suggested that SIN inhibits proliferation and promotes apoptosis of HCC cells by MARCH1-mediated AMPK/STAT3 signaling pathway. This study provides new support for SIN as a clinical anticancer drug and illustrates that targeting MARCH1 could be a novel treatment strategy in developing anticancer therapeutics.

## Introduction

Hepatocellular carcinoma (HCC) is a common malignancy worldwide. According to GLOBOCAN 2018, about 841,080 new cases of HCC are diagnosed globally and 781,631 deaths occur annually. Approximately 50% of HCC cases and deaths are reported in China (Bray et al., 2018). The current management, such as transarterial chemoembolization (TACE), systemic treatment, and targeted therapy are primary choices for patients with intermediate or advanced stage HCC (Marrero, 2006; Zhu, 2012). Advanced HCC is commonly treated with therapeutic agents, include sorafenib, adriamycin, and 5-fluorouracil; however, multi-drug resistance often develops and limits the success of treatment, resulting in poor prognoses (Grem, 2000; Shen et al., 2013; Kalyan et al., 2015). Thus, it is needed to understand the molecular mechanisms and develop novel therapeutic strategies in HCC treatment.

Natural products represent a considerable source of diverse bioactive chemicals. For example, more than 4,000 alkaloids represent a large and important group of natural products that display potent and highly varied biological activities. Extracts and compounds, particularly alkaloids, isolated from different parts of plants, have been used medically for centuries. Sinomenine (SIN) is an alkaloid with anti-rheumatic, anti-inflammatory, neuroprotective, and analgesic properties. SIN is used extensively for the treatment of rheumatoid arthritis and neuralgia in China (Qian et al., 2007; Zhu et al., 2016; Liu et al., 2018). Some experimental studies indicate that SIN is useful for the treatment of gastric carcinoma, lung cancer and breast cancer through regulating PI3K/AKT/Wnt, JAK/STAT3, JNK, and MEK/ERK signaling pathways (Jiang et al., 2016; Gao et al., 2019; Liu et al., 2019). SIN hydrochloride inhibits proliferation, induces apoptosis and causes cell cycle arrest in human HCC cells (Hong et al., 2013; Lu et al., 2013); however, the underlying mechanism of action of SIN in anti-HCC remains to be elucidated.

Membrane-associated RING-CH finger protein1 (MARCH1) is a member of the MARCH family of membrane-bound E3 ubiquitin ligases. MARCH1 induces the ubiquitination of MHC II in B cells to reduce MHC II surface expression and prevent antigen presentation (Matsuki et al., 2007). Further, loss of MARCH1 in B cells and dendritic cells (DCs) leads to increase MHC I surface expression and results in ineffective antigen presentation (Wilson et al., 2018). Previously, MARCH1 played an important role in immune system. Recent years, increasing attentions have been paid on the research of MARCH1 in progression and development of cancer. Meng Y et al. reported that silencing MARCH1 suppresses ovarian cancer through downregulating NF-κB and Wnt/β-catenin signaling pathways (Meng et al., 2016). Our Lab found that MARCH1 was over-expressed in HCC cells and tissues, and promoted the proliferation of HCC cells. MARCH1 downregulation induced by siRNA or drugs could inhibit the progression of HCC by down-regulating PI3K-AKT-β-catenin and PTEN/AKT signaling pathways (Xie et al., 2019a; Xie et al., 2019b; Dai et al., 2020). However, the molecular mechanism of SIN in inhibiting MARCH1 expression and its downstream signaling molecules is unclear.

AMPK/STAT3 signaling pathways are closely related to tumor biological functions, such as proliferation, and apoptosis (Lin et al., 2011; Jeon et al., 2012; De Veirman et al., 2019). It is reported that STAT3 signaling pathway participates in regulation of SIN against lung cancer and osteosarcoma cells (Jiang et al., 2016; Xie et al., 2016). However, whether SIN inhibits HCC by regulating the STAT3 signaling pathway has not been reported. In addition, AMPK signaling pathway has not been studied in anti-cancer of SIN. Therefore, we explored the role of SIN on AMPK/STAT3 signaling pathway in Hep3B and HepG2 cells. Furthermore, we assessed the involvement MARCH1 in regulating AMPK/STAT3 pathways by silencing MARCH1 in Hep3B and HepG2 cells. Our study demonstrated that SIN inhibits proliferation of HCC cells and promotes apoptosis by inhibiting MARCH1 expression and AMPK/STAT3 signaling pathway.

## Materials and Methods

### Cell Culture

Human liver cancer cell lines, HepG2 and Hep3B, were obtained from the Chinese Academy of Science (Shanghai, China). Cells were cultured in Dulbecco’s modified Eagle’s medium (DMEM) with high glucose (Hyclone, Logan, UT, United States), 10% fetal bovine serum (Gibco, Waltham, MA, United States), 100 units/ml penicillin and 100 μg/ml streptomycin (Solarbio, Beijing, China), and incubated at 37°C in a humified atmosphere with 5% CO_2_.

### Reagents and Antibodies

Sinomenine was purchased from Solarbio Life Sciences (#SS8560,Solarbio, Beijing, China), dissolved in DMSO and placed away from light at −20°C. Anti-MARCH1 antibody (#bs-9335R) was obtained from Bioss (Beijing, China). Anti-p-STAT3 (#ab32143), p-AMPK (#ab92701) and AMPK (#ab32047) antibodies were purchased from Abcam (Cambridge, United Kingdom). Anti-Bcl-2 (#12789-1-AP), CyclinB1 (#55004-1-AP), CyclinD1 (#26939-1-AP), Mcl-1 (#16225-1-AP), Bax (#50599-2-Ig), STAT3 (#10253-2-AP), GAPDH (#10494-1-AP) and Peroxidase-conjugated Affinipure Goat anti-Rabbit IgG (H + L) (#SA00001-2) antibodies were purchased from the Proteintech Group (Chicago, IL, United States).

### Transfection of SiRNA

SiRNA sequences of MARCH1 were designed and synthesized by Genepharma (Shanghai, China). The sequence is: MARCH1 siRNA-1: sense: 5′-CAG​GAG​GUC​UUG​UCU​UCA​UTT-3′; antisense: 5′-AUG​AAG​ACA​AGA​CCU​CCU​GTT-3′. MARCH1 siRNA-2: sense: 5′-GGU​AGU​GCC​UGU​ACC​ACA​ATT-3′; antisense: 5′-UUG​UGG​UAC​AGG​CAC​UAC​CTT-3′. Non-target siRNA: sense: 5-UUC​UCC​GAA​CGU​GUC​ACG​UTT-3′; antisense: 5′-ACG​UGA​CAC​GUU​CGG​AGA​ATT-3′. All siRNAs were dissolved in DEPC-treated water. The transfection was performed according to the manufacturer’s instructions. Cells were seeded in 6‐well culture dishes to reach about 50% confluence. Then, 50 nM siRNA or 5 μl Lipofectamine 2,000 (Invitrogen, Carlsbad, CA, United States) were added into 100 μl serum-free DMEM/High glucose medium and allowed to incubate at room temperature for 5 min. Lipofectamine 2,000 complex was mixed with the siRNA complex and allowed to stand at room temperature for 20 min. Finally, the mixture was added into each well and incubated in 37°C incubator for 5 h. Medium with penicillin-streptomycin was replaced with the transfection medium, and incubation was continued for 48 h.

### Western Blot

Cells were lysed in pre-cooled RIPA lysis buffer (#P0013B, Beyotime, Shanghai, China) containing proteasome and phosphatase inhibitors, then, incubated on ice for 40 min and centrifuged for 20 min at the speed of 12,000 rpm to acquire cell lysates. BCA Protein Assay Kit (#PC0020, Solarbio, Beijing, China) was used for protein quantification. Protein samples were boiled in SDS-PAGE sample loading buffer for 8 min at 99°C, and at least 20 μg proteins were separated in SDS-PAGE gels (#P1200, Solarbio, Beijing, China). After electrophoresis, the gels were transferred to PVDF membrane (#ISEQ00010, Immobilon^®^-PSQ PVDF 0.2 μm, Ireland). Membranes were then blocked at room temperature for 2.5 h in 5% skim milk prepared in tris buffered saline and Tween 20 (TBST, PH 7.4). Next, membranes were incubated with primary antibodies overnight at 4°C. Membranes were washed four times in TBST, and then incubated with secondary antibodies for 40 min at 37°C. Finally, membranes were again washed four times in TBST, and protein bands visualize with chemiluminescent reagent (#PWB-001S, Novland, Shanghai, China). Images were obtained by ChemiDocTM XRS+ (BIO-RAD, Hercules, CA, United States). Photoshop CS6 was used to quantify protein bands.

### CCK-8 Assay

Cells were inoculated into 96-well plates at 5,000 cells per well and incubated for 24 h at 37°C. Cells were then treated with SIN for 24 h. CCK-8 reagent (Biosharp, Beijing, China) was added to each well and reacted with cells for 1 h at 37°C following the manufacturer’s instructions. Light absorbance at 450 nm was measured by a microplate reader (SpectraMax M2, Molecular Devices, Shanghai, China).

### Colony Formation Assay

Cells were inoculated into 6-well plates at 8,000 cells each well. After 24 h incubation at 37°C, SIN was added, and incubation continued for 36 h. Subsequently, cells were incubated in complete medium without SIN for 10 days. Cells were fixed with methanol for 20 min and washed with phosphate buffer solution (PBS). Clones were stained with 0.1% crystal violet solution (#G1064, Solarbio, Beijing, China), washed by PBS, and dried. Finally, the number of clones was counted by Photoshop CS6 and colony-formation efficiency was calculated.

### Cell Proliferation Assay

Cell proliferation was evaluated by EdU assay using a commercially available kit (#C10310-1, Ribobio, Guangzhou, China). Cells were seeded in 96-well plates at 5,000 cells per well, and incubated for 24 h at 37°C before treating with SIN. After 24 h of drug treatment, cells were incubated with 50 μM EdU medium for 2 h. Cells were then immobilized with 4% polyoxymethylene for 30 min, treated with 2 mg/ml glycine for 5 min, and permeated by 0.5% TritonX-100 for 10 min. Cells were incubated in Apollo 567 for 30 min at room temperature in the dark and permeated by 0.5% TritonX-100 for 20 min. At last, cells were incubated with Hoechst 33,342 for 30 min at room temperature in the dark. PBS was used for washing between each step. Images were collected by a fluorescence microscope (Olympus TL4 photomicroscope, Japan).

### Cell Cycle Assay

A flow cytometer (Becton Dickinson FACSCantoTM II, Franklin Lakes, NJ, United States) was used to determine the phase of cell cycle arrest in cells treated with SIN. Cells were prepared following instructions included in the cell cycle detection kit (#CA002, SAB, Maryland, United States). Briefly, cells were detached from 6-well plates and fixed in pre-cooled 75% ethanol overnight at 4°C. Then, cells were collected and resuspended in PBS. Cells were treated with RNase for 30 min at 37°C, and stained with propidium iodide for 30 min at 4°C. Fluorescent intensity was measured within 1 h after staining.

### qRT-PCR

Total RNA was extracted from Hep3B and HepG2 cells using the TRIZOL reagents. The primer of human GAPDH and MARCH1 were purchased from Takara. GAPDH sequences: F: 5′-GCA​CCG​TCA​AGG​CTG​AGA​AC-3′; R: 5′-TGG​TGA​AGA​CGC​CAG​TGG​A-3'. MARCH1 sequences: F:5′-CTGCTGTGAGCTCTGCAAGTATGA-3′; R: 5′-TAC​GTG​GAA​TGT​GAC​AGA​GCA​GAA-3′. A reverse transcription kit (#RR047A, Takara) was used for reversed transcription into cDNA. Real-Time PCR used Tli RNaseH Plus (#RR820A, Takara). GAPDH and MARCH1 were amplified and measured by QuantStudio 3. PCR products were quantified using the 2^−ΔΔct^ method.

### Statistical Analysis

Data analysis was carried out using the GraphPad Prism 7 (GraphPad Software Inc, San Diego, United States). All experimental data are presented as the means ± SD from at least three independent experiments. Differences between the groups were assessed using Student’s t-test and ANOVA. *p* < 0.05 was considered significant.

## Results

### SIN Suppresses Expression of MARCH1 in HCC Cells

**FIGURE 1:**
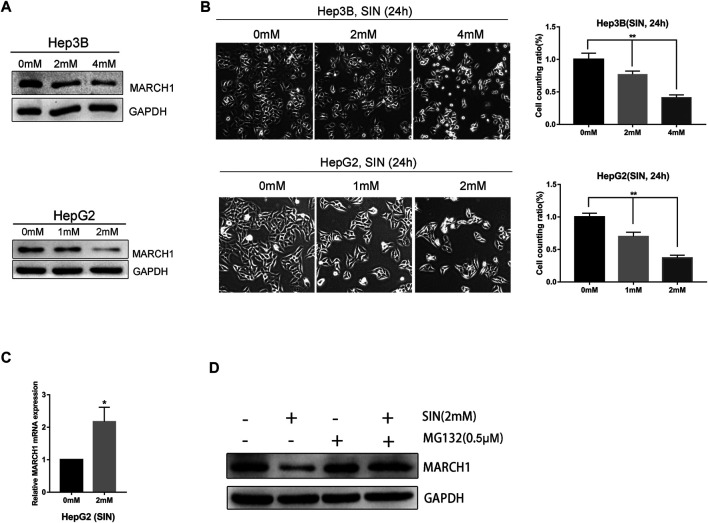
SIN downregulated MARCH1 expression in HCC cells. (A) Western Blotting on the protein expression of MARCH1 in Hep3B and HepG2 cells treated with SIN. **(B)** The morphology of Hep3B and HepG2 cells treated with different concentrations of SIN for 24 h, the number of cells counted and analyzed. 0 mM as control. **(C)** qRT-PCR on the mRNA expression of MARCH1 in HepG2 cells treated with SIN. **(D)** Western Blotting on the protein expression of MARCH1 HepG2 cells treated with MG132 (0.5μΜ) and with or without SIN (2 mM). All data are means ± SD of results from three experiments. * represents *p* < 0.05, ** represents *p* < 0.01.

The Hep3B and HepG2 cells were treated with different concentrations of SIN for 24 h and its antitumor effect was evaluated. We found that the expression of MARCH1 was down-regulated in both Hep3B and HepG2 cells following SIN treatment in a dose-dependent manner, as measured by Western Blotting (Figure 1A). Microscopically, the number of cells decreased, some cell bodies shrank and cytoplasm condensed, and some cells became round and bright after exposure to SIN (Figure 1B). We performed qPCR to evaluate the mRNA expression of MARCH1. Interestingly, the mRNA level of MARCH1 was slightly increased in HepG2 cells (Figure 1C). HepG2 cells were then treated with SIN with or without MG132, a proteasome inhibitor, to explore the reason of MARCH1 protein down-regulation. Low protein expression of MARCH1 caused by SIN was rescued by MG132 treatment (Figure 1D). These results suggested that SIN reduces the expression of MARCH1 by proteasome-mediated protein degradation.

### Low Expression of MARCH1 Attenuates Phosphorylation of AMPK and STAT3

**FIGURE 2:**
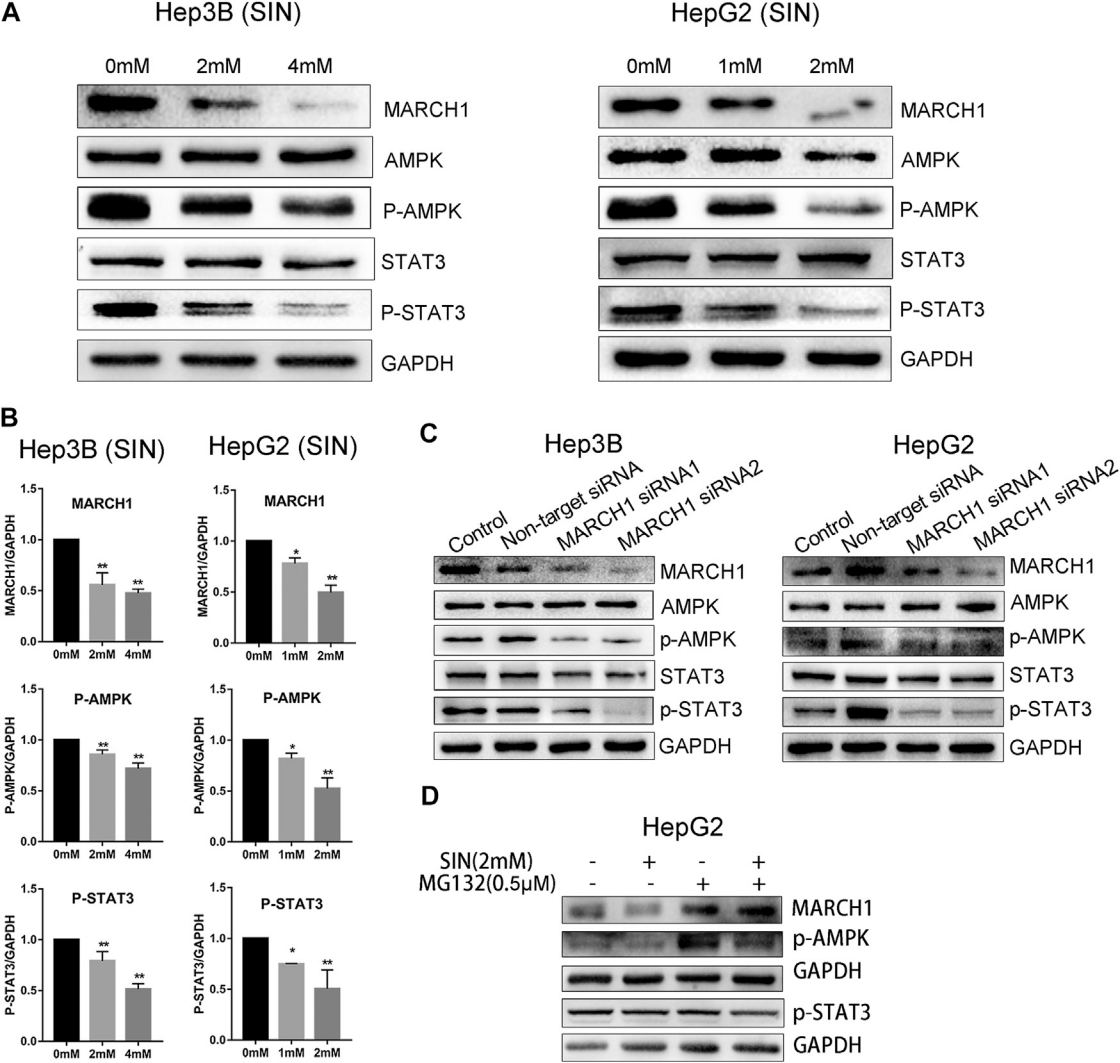
SIN decreased phosphorylation of AMPK and STAT3 *via* inhibiting MARCH1. **(A)** The expression of MARCH1 and AMPK/STAT3 signaling molecules detected by Western Blotting in Hep3B and HepG2 cells treated with SIN for 24 h. **(B)** The protein expression of MARCH1, p-AMPK and p-STAT3 quantified by measuring the gray band value using photoshop CS6. GAPDH was used for normalization. 0 mM as control. **(C)** The protein expression of AMPK, p-AMPK, STAT3 and p-STAT3 after MARCH1 knockdown in Hep3B and HepG2 cells. **(D)** p-AMPK and p-STAT3 expression were detected when HepG2 cells were treated with SIN and MG132. All data are means ± SD of results from three experiments. * represents *p* < 0.05, ** represents *p* < 0.01.

We used Western Blotting to measure expression of AMPK/STAT3 signaling molecules. SIN treatment decreases the expression of p-AMPK and p-STAT3 in Hep3B and HepG2 cells (Figure 2A). Expression of these proteins were normalized using GAPDH as a reference, and statistical analysis on these signaling molecules in Hep3B and HepG2 are provided in Figure 2B. SiRNA was used to knockdown MARCH1 in these cells to confirm its involvement in inhibiting AMPK/STAT3 signaling pathway. The results showed that phosphorylation of AMPK and STAT3 decreases after MARCH1 knockdown in Hep3B and HepG2 cells (Figure 2C). These results demonstrated that SIN down-regulates phosphorylation of AMPK and STAT3 through down-regulating expression of MARCH1. To solidify this conclusion, the p-AMPK and p-STAT3 expression were detected when HepG2 cells were treated with SIN combined with or without MG132. The results showed that p-AMPK expression was partly rescued when MARCH1 was rescued by MG132. But the rescue effect of MG132 to p-STAT3 was not obvious. (Figure 2D and Supplementary Material).

### Sinomenine Induces HCC Cell Apoptosis

**FIGURE 3:**
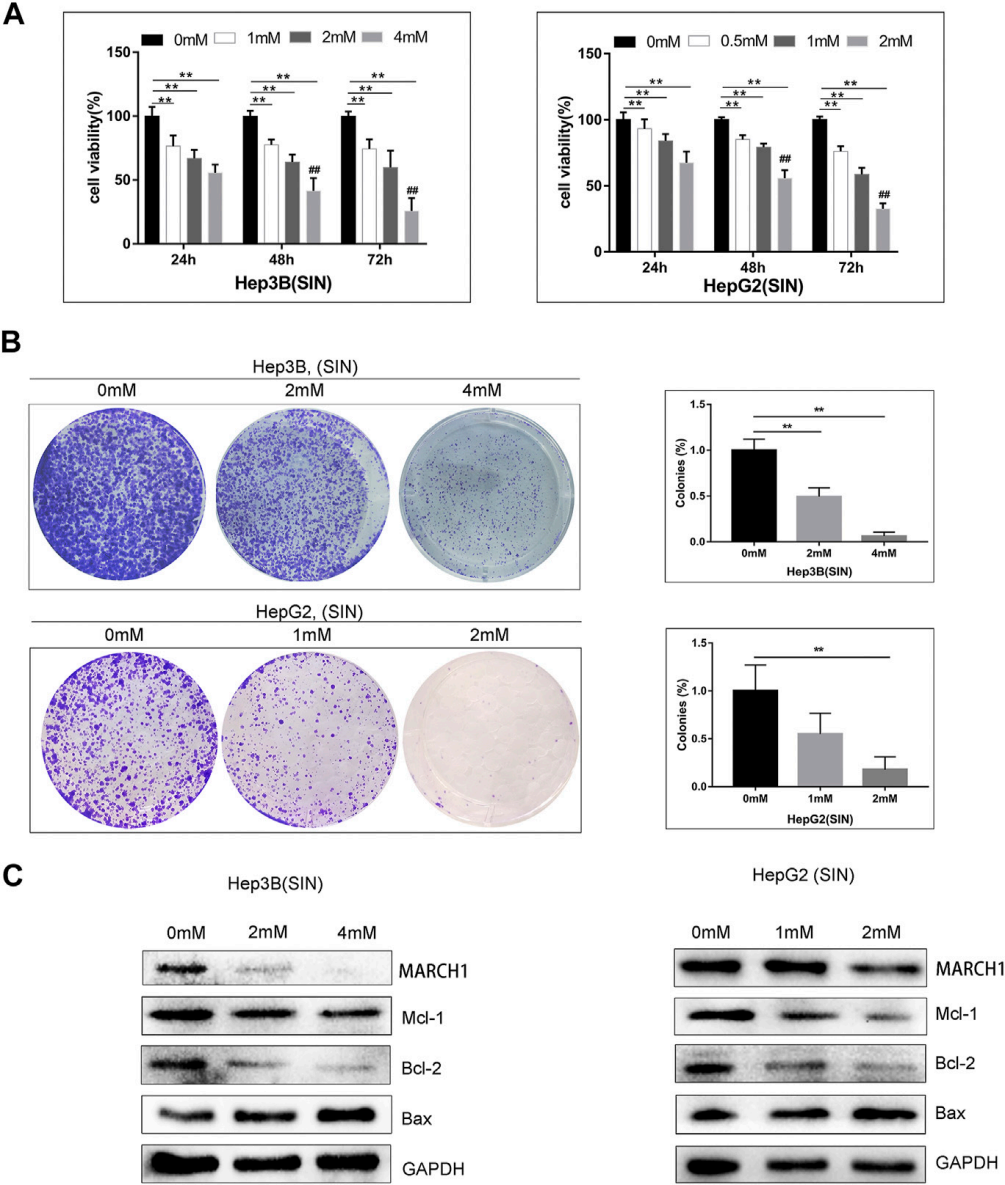
SIN induced HCC cells apoptosis. **(A)** CCK-8 assay on the viability of Hep3B and HepG2 cells treated with SIN for 24, 48, and 72 h. 0 mM as control. **(B)** The Hep3B and HepG2 cells was treated with SIN (Hep3B: 0, 2, 4 mM; HepG2: 0, 1, 2 mM) for 36 h, and then incubated in fresh medium without SIN for 10 days. The colonies were fixed and stained. 0 mM as control. **(C)** The apoptosis-related signaling molecules Mcl-1, Bax and Bcl-2 tested in Hep3B and HepG2 cells. All data are means ± SD of results from three experiments. ** represents *p* < 0.01; ## indicates *p* < 0.01.

AMPK/STAT3 signaling pathway is involved in regulating tumor cell proliferation, apoptosis, and cell cycle. CCK-8 and colony-formation assays were used to evaluate whether SIN affects these biological behaviors of these HCC cells. It was found that SIN intervention suppresses the growth of Hep3B and HepG2 cells in a time and dose-dependent manner (Figure 3A). In addition, cells treated with different concentrations of SIN for 36 h were cultured for 10 days after treatment of SIN. The number and the size of colonies declined in a dose-dependent manner in SIN treated cells (Figure 3B). Moreover, the expression of apoptosis-related proteins was examined by Western Blotting. The result showed that the expression of pro-apoptotic protein, Bax, was elevated and anti-apoptotic molecules including, Mcl-1 and Bcl-2 were down-regulated in Hep3B and HepG2 cells. This result is in consistent with the previous study (Lu et al., 2013). (Figure 3C).

### Sinomenine Causes HCC Cell Cycle Arrest

**FIGURE 4:**
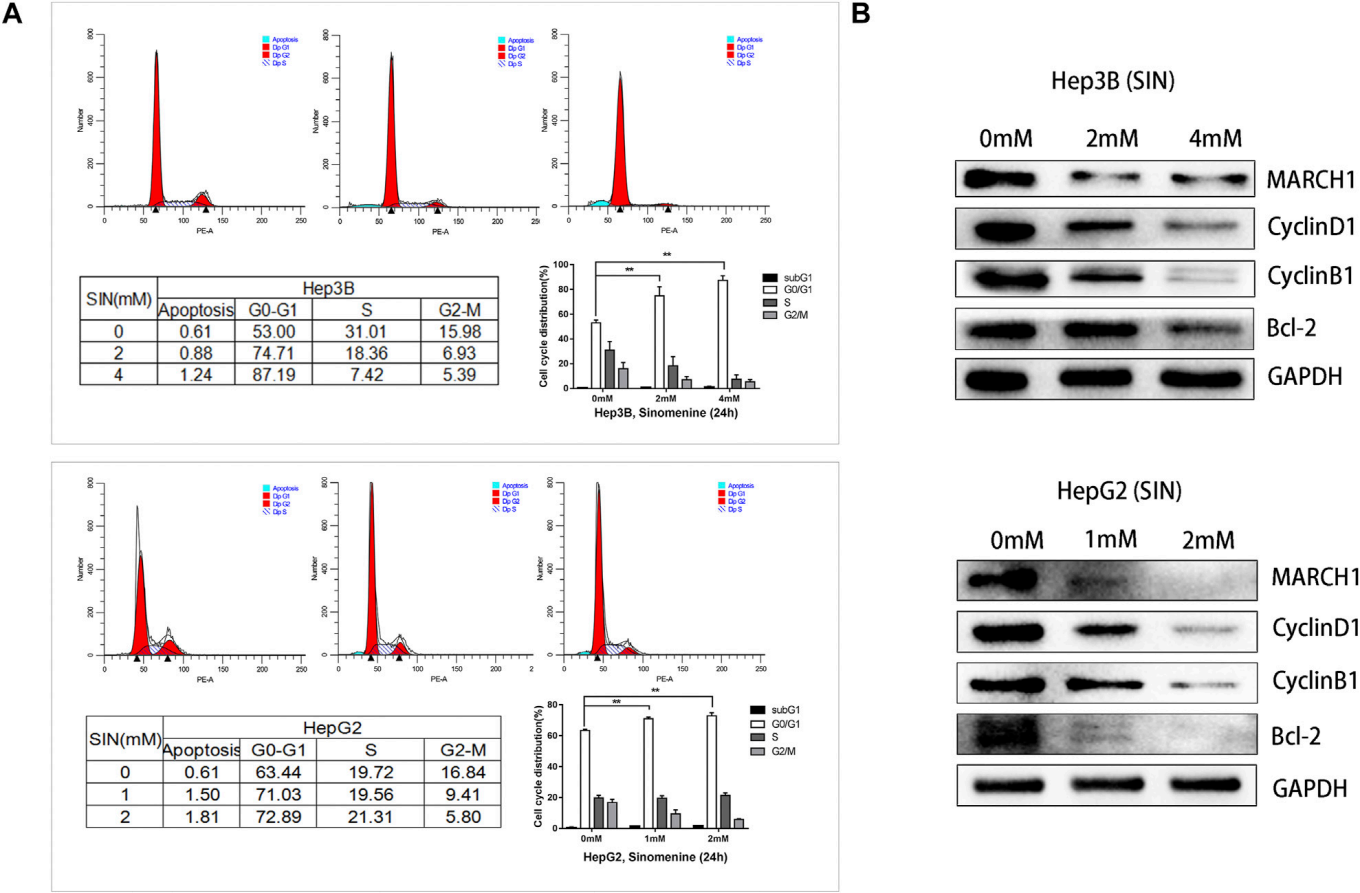
SIN Causes HCC Cell Cycle Arrest. **(A)** Flow cytometric analysis on cell cycle of Hep3B and HepG2 cells treated with or without SIN, and data are presented in tables and statistical graphs. **(B)** Cell cycle-related proteins including MARCH1, CyclinD1, CyclinB1 and Bcl-2 measured by Western Blotting. 0 mM as control.

Flow cytometric analysis was performed to pinpoint the phase of cell cycle arrest following SIN treatment. HepG2 and Hep3B cells exposed to different concentrations of SIN showed G0/G1 phase arrest (Figure 4A). Furthermore, we found that the expression of cell cycle-related proteins, CyclinD1, CyclinB1, and Bcl-2 were down-regulated in both Hep3B and HepG2 cells (Figure 4B). These results indicated that SIN might induce cells cycle arrest by down-regulating the expression of these cell cycle associated proteins.

### Sinomenine Suppresses HCC Cell Proliferation by Inhibiting MARCH1

**FIGURE 5:**
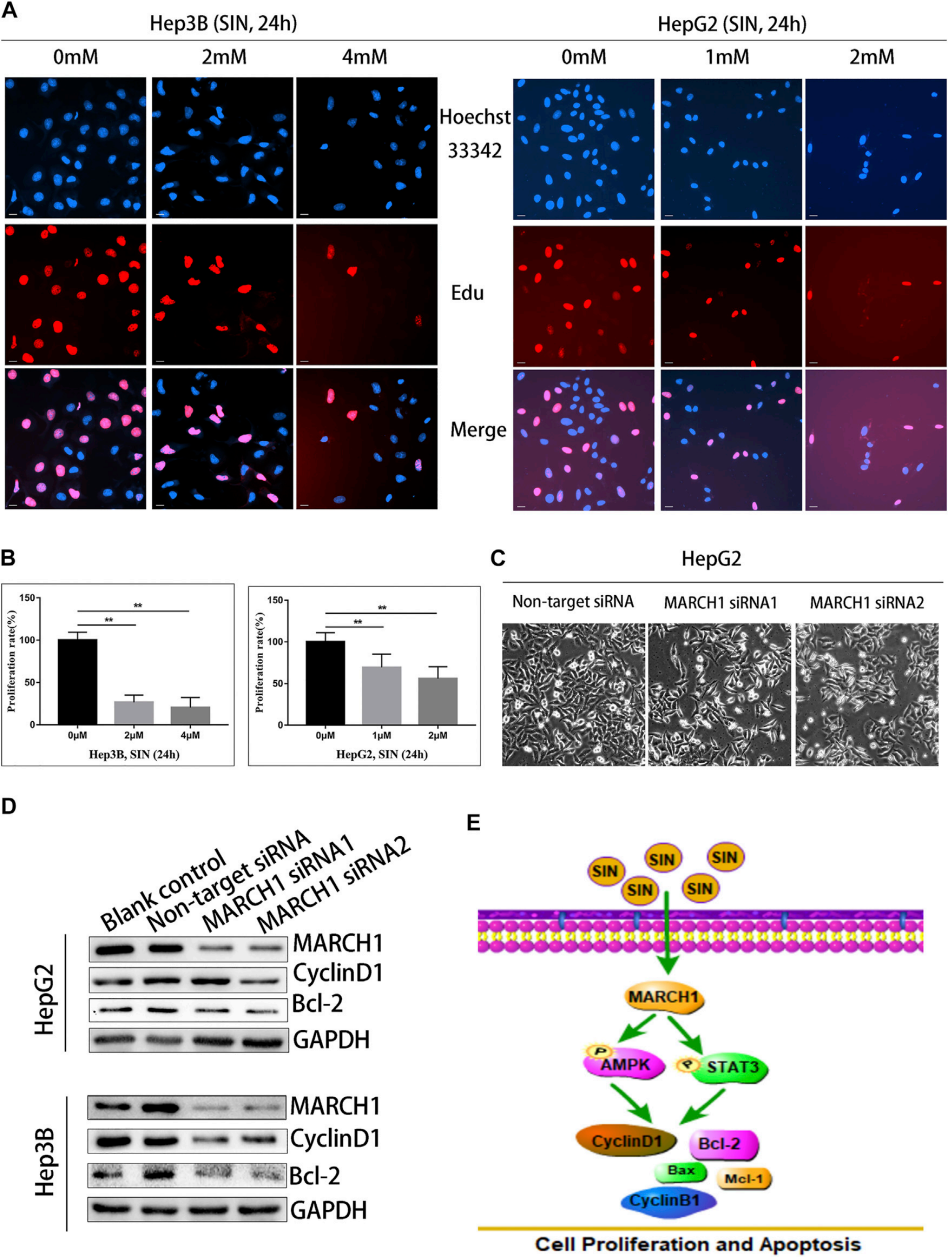
SIN suppressed the proliferation of HCC cells by inhibiting MARCH1. **(A)** The EdU assay. The Hep3B and HepG2 cells were treated with SIN (Hep3B: 0, 2, 4 mM HepG2: 0, 1, 2 mM) for 24 h. The number of proliferative nuclei were counted. Bar, 20 μm. 0 mM as control. **(B)** The statistical graphs of different concentration of SIN on HCC cells inhibition. **(C)** The morphology of cells after silencing MARCH1 in HepG2 cells. **(D)** The expression of CyclinD1 and Bcl-2 detected after MARCH1 knockdown using siRNA in Hep3B and HepG2 cells. **(E)** The schemm shows that SIN regulates MARCH1-mediated AMPK/STAT3 signaling to affect cell proliferation and apoptosis. All data are means ± SD of results from three experiments. ** represents *p* < 0.01.

The EdU assay was implemented to assess whether SIN inhibits HCC cell proliferation. Nuclei were stained with EdU and Hoechst reagents. The result showed that the number of EdU positive cells were reduced compared to untreated control cells. (Figures 5A,B). To evaluate whether MARCH1 participates in the inhibition of SIN on HCC cell proliferation, MARCH1 was knocked down using siRNA in HepG2 cells. The result showed that the surviving HepG2 cells were significantly reduced in knockdown cells compared to control cells (Figure 5C). Furthermore, MARCH1 knockdown down-regulated the protein expression of CyclinD1 and Bcl-2 (Figure 5D). These results revealed that SIN suppresses cell proliferation and promotes apoptosis of HCC cells by inhibiting MARCH1. All results in this study indicated that the effect of SIN in inhibiting proliferation and inducing apoptosis in HCC cells is based on inhibition of MARCH1, resulting in down-regulating AMPK/STAT3 signaling pathway and downstream molecules, CyclinD1, CyclinB1, Mcl-1, Bax, and Bcl-2 (Figure 5E).

## Discussion

In the present study, we revealed that SIN, one of the alkaloid natural products, possesses potential anti-cancer effect. We found that SIN induces cell apoptosis and arrests the proliferation of HCC cells. These activities are partly dependent on down-regulation of MARCH1 expression. Interestingly, SIN down-regulated the MARCH1 protein expression but increased MARCH1 transcription in HepG2 cells. MG132, as a proteasome inhibitor Dai et al. (2020), rescues MARCH1 protein expression in HepG2 cells after SIN treatment. Therefore, SIN-induced MARCH1 down-regulation through proteasomal degradation. Moreover, treatment of Hep3B and HepG2 cells with SIN or siRNA of MARCH1 impaired the phosphorylation of AMPK and STAT3.

SIN is a common plant-derived alkaloid, and some experimental studies recognize its anticancer activities. SIN inhibits proliferation, invasion, and migration and promotes apoptosis in cancer cells by modulating signaling pathways and microRNAs, such as PI3K/AKT/Wnt signaling pathway, SHh pathway, and miR-23a (Song et al., 2018; Liu et al., 2019; Xu et al., 2019). We uncover that SIN induces HCC cell apoptosis and inhibits proliferation *via* the AMPK pathway. AMP-activated protein kinase (AMPK) is known as a sensor of the energy status of cells. It regulates the whole-body as well as cellular energy balance by monitoring the levels of AMP, ADP and ATP (Hardie et al., 2012; Hardie, 2015; Ross et al., 2016). AMPK inhibits cancer cell growth and tumorigenesis *via* regulating mitochondria-mediated metabolism and the LKB1-AMPK pathway. Loss of AMPK signaling works with oncogenic Myc to enhance tumorigenesis (Shackelford and Shaw, 2009; Faubert et al., 2013; Jiang et al., 2019). AMPK is a tumor suppressor; it is pivotal in cell survival during metabolic stress that can often occur under pathological conditions, such as tumor. In these situations, AMPK promotes cell survival in both normal and tumor cells (Chhipa et al., 2018). Many reports indicate that AMPK activation promotes survival of tumor cells *via* NADPH, AMPK–CREB1, and AMPK-mTOR-Akt/ERK signaling pathways (Jeon et al., 2012; Gao et al., 2016; Chhipa et al., 2018). However, the role of AMPK in SIN anticancer treatment is not reported. This study is the first one to show that SIN reduces the activity of p-AMPK in HCC cells. We also observe that SIN decreases expression of Bcl-2 in Hep3B and HepG2 cells. Notably, activation of AMPK-induced tumor cell survival is associated with Bcl-2. Kim De Veirman indicated that AMPK phosphorylation induces myeloid-derived suppressor cells that promote multiple myeloma progression. These cells cause immuno-suppression and induction of angiogenesis that are associated with an increase in anti-apoptotic factors Mcl-1 and Bcl-2 (De Veirman et al., 2019). Also, Bcl-2 is linked to AMPK-mediated protection against apoptosis of cardiomyocytes (Accordi et al., 2013). Thus, these studies support our findings that SIN promotes apoptosis and inhibits proliferation in HCC cells by inhibiting the AMPK-Bcl-2 pathway.

Furthermore, STAT3 signaling also participates in the anticancer activity of SIN. STAT3 signaling is crucial for the signal transduction cascade, in which cancer cells sense and adapt to a variety of environmental stimuli. STAT3 is also important to cell activities, such as proliferation, apoptosis and is a prominent target for cancer therapy (Corvinus et al., 2005; Chai et al., 2016; Huynh et al., 2019). Many studies show that inactivation of STAT3 pathway inhibits tumor cell proliferation, angiogenesis and metastasis (Chen et al., 2012; Chai et al., 2016). Previous experimental studies showed that SIN exerts its anticancer effect in prostate cell carcinoma and human osteosarcoma cells *via* inactivating JAK/STAT and CXCR-4-STAT3 signaling pathways (Xie et al., 2016; Xu et al., 2019). In this study, we demonstrated that SIN down-regulated the activity of p-STAT3 in Hep3B and HepG2 cells. Moreover, STAT3 signaling pathway positively regulates CyclinD1, Survivin, Bcl-2, Bcl-XL, and Mcl-1 to facilitate cancer cell cycle progression (Epling-Burnette et al., 2001; Lin et al., 2011). Thus, SIN exerts anti-apoptotic and proliferative function in HCC cells by regulating CyclinD1 and Bcl-2 through STAT3 signaling pathway. Also, the reduction of CyclinD1 and CyclinB1 in HCC cells treated with SIN is in agreement with a previous study, in which CyclinD1 and CyclinB1 down-regulation promoted cells exit from G2/M and enter into G0/G1 phase (Wang et al., 2018).

Silencing MARCH1 was achieved by transfection of siRNA-MARCH1 sequences into Hep3B and HepG2 cells. Western Blotting analysis on the expression of AMPK/STAT3 signaling related proteins showed that phosphorylation of AMPK and STAT3, and the expression of CyclinD1 and Bcl-2 in HCC cells were significantly down-regulated by MARCH1 knockdown. Thus, SIN down-regulates phosphorylation of AMPK and STAT3 through down-regulating expression of MARCH1 to inhibit HCC cells proliferation. In addition, p-AMPK expression was partly incresesd when down-regulation of MARCH1 induced by SIN was rescued by MG132. This partially confirms the above conclusion. But p-STAT3 expression was not significant change. This suggested that STAT3 signaling pathway may be regulated by more than just MARCH1. There may be another molecule mechanism to regulate STAT3 signaling pathway when HCC cells were treated with SIN, and the underlying molecular mechanisms need to be further explored.

Taken together, this study suggests that SIN treatment decreases MARCH1 expression, resulting in inhibiting AMPK/STAT3 signaling pathway, therefore inhibiting proliferation and enhancing apoptosis in HCC cells. This study provides a rationale for using SIN for the treatment of HCC and supports the application of SIN as a clinical anticancer drug.

## Data Availability

The original contributions presented in the study are included in the article/Supplementary Material, further inquiries can be directed to the corresponding authors.

## Abbreviations

HCC, hepatocellular carcinoma; SIN, sinomenin; MARCH1, membrane-associated RING-CH finger protein on; eGLOBOCAN, The Global Caner Observatory, CANCER TODAY; SiRNA, small interfering RNA; SDS-PAGE, sodium dodecyl sulfate polyacrylamide gel electrophoresis; PBS, phosphate buffer solution.

## References

[B1] Accordi B. Galla L. Milani G. Curtarello M. Serafin V. Lissandron V. (2013). AMPK Inhibition Enhances Apoptosis in MLL-Rearranged Pediatric B-Acute Lymphoblastic Leukemia Cells. Leukemia 27 (5), 1019–1027. 10.1038/leu.2012.338 23228943

[B2] Bray F. Ferlay J. Soerjomataram I. Siegel R. L. Torre L. A. Jemal A. (2018). Global Cancer Statistics 2018: GLOBOCAN Estimates of Incidence and Mortality Worldwide for 36 Cancers in 185 Countries. CA: A Cancer J. Clinicians 68 (6), 394–424. 10.3322/caac.21492 30207593

[B3] Chai E. Z. P. Shanmugam M. K. Arfuso F. Dharmarajan A. Wang C. Kumar A. P. (2016). Targeting Transcription Factor STAT3 for Cancer Prevention and Therapy. Pharmacol. Ther. 162, 86–97. 10.1016/j.pharmthera.2015.10.004 26478441

[B4] Chen J. Wang J. Lin L. He L. Wu Y. Zhang L. (2012). Inhibition of STAT3 Signaling Pathway by Nitidine Chloride Suppressed the Angiogenesis and Growth of Human Gastric Cancer. Mol. Cancer Ther. 11 (2), 277–287. 10.1158/1535-7163.mct-11-0648 22203730

[B5] Chhipa R. R. Fan Q. Anderson J. Muraleedharan R. Huang Y. Ciraolo G. (2018). AMP Kinase Promotes Glioblastoma Bioenergetics and Tumour Growth. Nat. Cel Biol. 20 (7), 823–835. 10.1038/s41556-018-0126-z PMC6113057 29915361

[B6] Corvinus F. M. Orth C. Moriggl R. Tsareva S. A. Wagner S. Pfitzner E. B. (2005). Persistent STAT3 Activation in Colon Cancer Is Associated with Enhanced Cell Proliferation and Tumor Growth. Neoplasia 7, 545–555. 10.1593/neo.04571 16036105 PMC1501283

[B7] Dai H. Li M. Yang W. Sun X. Wang P. Wang X. (2020). Resveratrol Inhibits the Malignant Progression of Hepatocellular Carcinoma *via* MARCH1-Induced Regulation of PTEN/AKT Signaling. Aging 12 (12), 11717–11731. 10.18632/aging.103338 32530437 PMC7343503

[B8] De Veirman K. Menu E. Maes K. De Beule N. De Smedt E. Maes A. (2019). Myeloid-derived Suppressor Cells Induce Multiple Myeloma Cell Survival by Activating the AMPK Pathway. Cancer Lett. 442, 233–241. 10.1016/j.canlet.2018.11.002 30419344

[B9] Epling-Burnette P. K. Liu J. H. Catlett-Falcone R. Turkson J. Oshiro M. Kothapalli R. (2001). Inhibition of STAT3 Signaling Leads to Apoptosis of Leukemic Large Granular Lymphocytes and Decreased Mcl-1 Expression. J. Clin. Invest. 107 (3), 351–362. 10.1172/jci9940 11160159 PMC199188

[B10] Faubert B. Boily G. Izreig S. Griss T. Samborska B. Dong Z. (2013). AMPK Is a Negative Regulator of the Warburg Effect and Suppresses Tumor Growth *In Vivo* . Cel Metab. 17 (1), 113–124. 10.1016/j.cmet.2012.12.001 PMC3545102 23274086

[B11] Gao G. Liang X. Ma W. (2019). Sinomenine Restrains Breast Cancer Cells Proliferation, Migration and Invasion *via* Modulation of miR-29/PDCD-4 axis. Artif. Cell Nanomedicine, Biotechnol. 47 (1), 3839–3846. 10.1080/21691401.2019.1666861 31556312

[B12] Gao M. Kong Q. Hua H. Yin Y. Wang J. Luo T. (2016). AMPK-mediated Up-Regulation of mTORC2 and MCL-1 Compromises the Anti-cancer Effects of Aspirin. Oncotarget 7 (13), 16349–16361. 10.18632/oncotarget.7648 26918349 PMC4941319

[B13] Grem J. L. (2000). 5-Fluorouracil: Forty-Plus and Still Ticking. A Review of its Preclinical and Clinical Development. Invest. New Drugs 18 (4), 299–313. 10.1023/a:1006416410198 11081567

[B14] Hardie D. G. (2015). AMPK: Positive and Negative Regulation, and its Role in Whole-Body Energy Homeostasis. Curr. Opin. Cel Biol. 33, 1–7. 10.1016/j.ceb.2014.09.004 25259783

[B15] Hardie D. G. Ross F. A. Hawley S. A. (2012). AMPK: a Nutrient and Energy Sensor that Maintains Energy Homeostasis. Nat. Rev. Mol. Cel Biol. 13 (4), 251–262. 10.1038/nrm3311 PMC5726489 22436748

[B16] Hong Y. Yang J. Shen X. Zhu H. Sun X. Wen X. (2013). Sinomenine Hydrochloride Enhancement of the Inhibitory Effects of Anti-transferrin Receptor Antibody-dependent on the COX-2 Pathway in Human Hepatoma Cells. Cancer Immunol. Immunother. 62 (3), 447–454. 10.1007/s00262-012-1337-y 22941037 PMC11028739

[B17] Huynh J. Chand A. Gough D. Ernst M. (2019). Therapeutically Exploiting STAT3 Activity in Cancer - Using Tissue Repair as a Road Map. Nat. Rev. Cancer 19 (2), 82–96. 10.1038/s41568-018-0090-8 30578415

[B18] Jeon S.-M. Chandel N. S. Hay N. (2012). AMPK Regulates NADPH Homeostasis to Promote Tumour Cell Survival during Energy Stress. Nature 485 (7400), 661–665. 10.1038/nature11066 22660331 PMC3607316

[B19] Jiang S. Gao Y. Hou W. Liu R. Qi X. Xu X. (2016). Sinomenine Inhibits A549 Human Lung Cancer Cell Invasion by Mediating the STAT3 Signaling Pathway. Oncol. Lett. 12 (2), 1380–1386. 10.3892/ol.2016.4768 27446441 PMC4950784

[B20] Jiang S. Wang Y. Luo L. Shi F. Zou J. Lin H. (2019). AMP‐activated Protein Kinase Regulates Cancer Cell Growth and Metabolism *via* Nuclear and Mitochondria Events. J. Cel Mol Med 23 (6), 3951–3961. 10.1111/jcmm.14279 PMC6533503 30993829

[B21] Kalyan A. Nimeiri H. Kulik L. (2015). Systemic Therapy of Hepatocellular Carcinoma. Clin. Liver Dis. 19 (2), 421–432. 10.1016/j.cld.2015.01.009 25921671

[B22] Lin L. Liu A. Peng Z. Lin H.-J. Li P.-K. Li C. (2011). STAT3 Is Necessary for Proliferation and Survival in colon Cancer-Initiating Cells. Cancer Res. 71 (23), 7226–7237. 10.1158/0008-5472.can-10-4660 21900397 PMC4295768

[B23] Liu W. Zhang Y. Zhu W. Ma C. Ruan J. Long H. (2018). Sinomenine Inhibits the Progression of Rheumatoid Arthritis by Regulating the Secretion of Inflammatory Cytokines and Monocyte/Macrophage Subsets. Front. Immunol. 9 (9), 2228. 10.3389/fimmu.2018.02228 30319663 PMC6168735

[B24] Liu Y. Liu C. Tan T. Li S. Tang S. Chen X. (2019). Sinomenine Sensitizes Human Gastric Cancer Cells to Cisplatin through Negative Regulation of PI3K/AKT/Wnt Signaling Pathway. Anticancer Drugs. 30 (10), 983–990. 10.1097/cad.0000000000000834 31609766 PMC6824511

[B25] Lu X.-L. Zeng J. Chen Y.-L. He P.-M. Wen M.-X. Ren M.-D. (2013). Sinomenine Hydrochloride Inhibits Human Hepatocellular Carcinoma Cell Growth *In Vitro* and *In Vivo*: Involvement of Cell Cycle Arrest and Apoptosis Induction. Int. J. Oncol. 42 (1), 229–238. 10.3892/ijo.2012.1704 23165705

[B26] Marrero J. A. (2006). Hepatocellular Carcinoma. Curr. Opin. Gastroenterol. 22 (3), 248–253. 10.1097/01.mog.0000218961.86182.8c 16550039

[B27] Matsuki Y. Ohmura-Hoshino M. Goto E. Aoki M. Mito-Yoshida M. Uematsu M. (2007). Novel Regulation of MHC Class II Function in B Cells. EMBO J. 26 (3), 846–854. 10.1038/sj.emboj.7601556 17255932 PMC1794403

[B28] Meng Y. Hu J. Chen Y. Yu T. Hu L. (2016). Silencing MARCH1 Suppresses Proliferation, Migration and Invasion of Ovarian Cancer SKOV3 Cells *via* Downregulation of NF-Κb and Wnt/β-Catenin Pathways. Oncol. Rep. 36 (5), 2463–2470. 10.3892/or.2016.5076 27633480 PMC5055210

[B29] Qian L. Xu Z. Zhang W. Wilson B. Hong J. S. Flood P. M. (2007). Sinomenine, a Natural Dextrorotatory Morphinan Analog, Is Anti-inflammatory and Neuroprotective through Inhibition of Microglial NADPH Oxidase. J. Neuroinflammation 19 (4), 23. 10.1186/1742-2094-4-23 PMC2064906 17880684

[B30] Ross F. A. MacKintosh C. Hardie D. G. (2016). AMP-activated Protein Kinase: a Cellular Energy Sensor that Comes in 12 Flavours. FEBS J. 283 (16), 2987–3001. 10.1111/febs.13698 26934201 PMC4995730

[B31] Shackelford D. B. Shaw R. J. (2009). The LKB1-AMPK Pathway: Metabolism and Growth Control in Tumour Suppression. Nat. Rev. Cancer 9 (8), 563–575. 10.1038/nrc2676 19629071 PMC2756045

[B32] Shen Y. C. Lin Z. Z. Hsu C. H. Hsu C. Shao Y. Y. Cheng A. L. (2013). Clinical Trials in Hepatocellular Carcinoma: an Update. Liver Cancer 2 (3-4), 345–364. 10.1159/000343850 24400222 PMC3881316

[B33] Song L. Liu D. Zhao Y. He J. Kang H. Dai Z. (2018). Sinomenine Reduces Growth and Metastasis of Breast Cancer Cells and Improves the Survival of Tumor-Bearing Mice through Suppressing the SHh Pathway. Biomed. Pharmacother. 98, 687–693. 10.1016/j.biopha.2017.12.065 29304494

[B34] Wang Z. Wang Y. Wang S. Meng X. Song F. Huo W. (2018). Coxsackievirus A6 Induces Cell Cycle Arrest in G0/G1 Phase for Viral Production. Front Cel Infect Microbiol 8, 279. 10.3389/fcimb.2018.00279 PMC6104138 30159255

[B35] Wilson K. R. Liu H. Healey G. Vuong V. Ishido S. Herold M. J. (2018). MARCH1-mediated Ubiquitination of MHC II Impacts the MHC I Antigen Presentation Pathway. PLoS One. 13 (7), e0200540. 10.1371/journal.pone.0200540 30001419 PMC6042767

[B36] Xie L. Dai H. Li M. Yang W. Yu G. Wang X. (2019). MARCH1 Encourages Tumour Progression of Hepatocellular Carcinoma *via* Regulation of PI3K‐AKT‐β‐catenin Pathways. J. Cel Mol Med. 23 (5), 3386–3401. 10.1111/jcmm.14235 PMC6484336 30793486

[B37] Xie L. Li M. Liu D. Wang X. Wang P. Dai H. (2019). Secalonic Acid-F, a Novel Mycotoxin, Represses the Progression of Hepatocellular Carcinoma *via* MARCH1 Regulation of the PI3K/AKT/β-catenin Signaling Pathway. Molecules 24 (3), 393. 10.3390/molecules24030393 PMC6385111 30678274

[B38] Xie T. Ren H.-Y. Lin H.-Q. Mao J.-P. Zhu T. Wang S.-D. (2016). Sinomenine Prevents Metastasis of Human Osteosarcoma Cells *via* S Phase Arrest and Suppression of Tumor-Related Neovascularization and Osteolysis through the CXCR4-STAT3 Pathway. Int. J. Oncol. 48 (5), 2098–2112. 10.3892/ijo.2016.3416 26983669

[B39] Xu F. Li Q. Wang Z. Cao X. (2019). Sinomenine Inhibits Proliferation, Migration, Invasion and Promotes Apoptosis of Prostate Cancer Cells by Regulation of miR-23a. Biomed. Pharmacother. 112, 108592. 10.1016/j.biopha.2019.01.053 30784907

[B40] Zhu A. X. (2012). Molecularly Targeted Therapy for Advanced Hepatocellular Carcinoma in 2012: Current Status and Future Perspectives. Semin. Oncol. 39 (4), 493–502. 10.1053/j.seminoncol.2012.05.014 22846866

[B41] Zhu Q. Sun Y. Mao L. Liu C. Jiang B. Zhang W. (2016). Antinociceptive Effects of Sinomenine in a Rat Model of Postoperative Pain. Br. J. Pharmacol. 173 (10), 1693–1702. 10.1111/bph.13470 26915970 PMC4842916

