# Supplementary figures and images for: Sinomenine Suppresses Development of Hepatocellular Carcinoma Cells via Inhibiting MARCH1 and AMPK/STAT3 Signaling Pathway
Source: Front Mol Biosci. 2021 Jun 10;8:684262. doi: 10.3389/fmolb.2021.684262 (PMC8222788; doi:10.3389/fmolb.2021.684262)

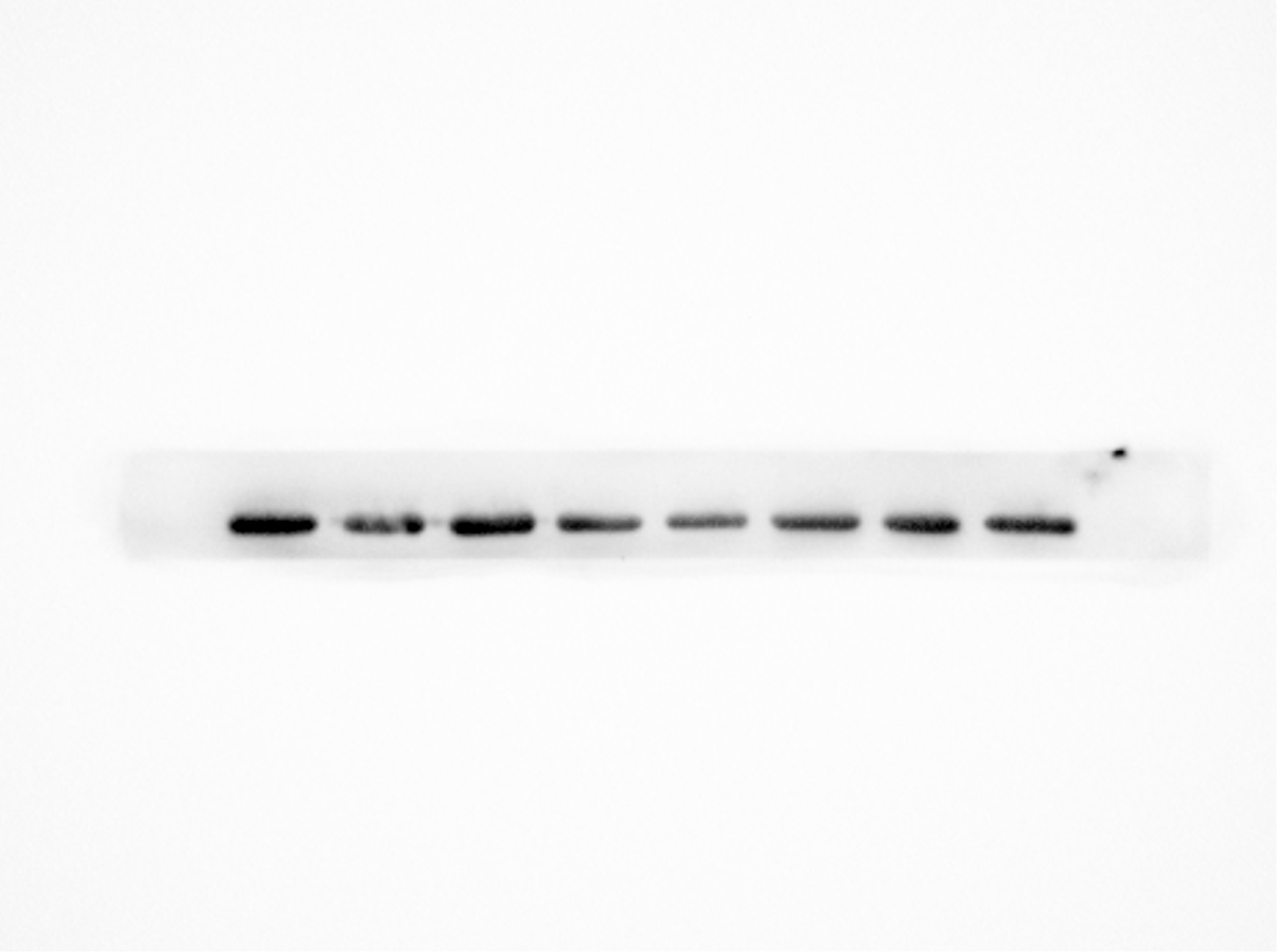

Supplement: Supplementary file 1 [file DataSheet1.ZIP › original data(supplement)/GAPDH-1.tif]

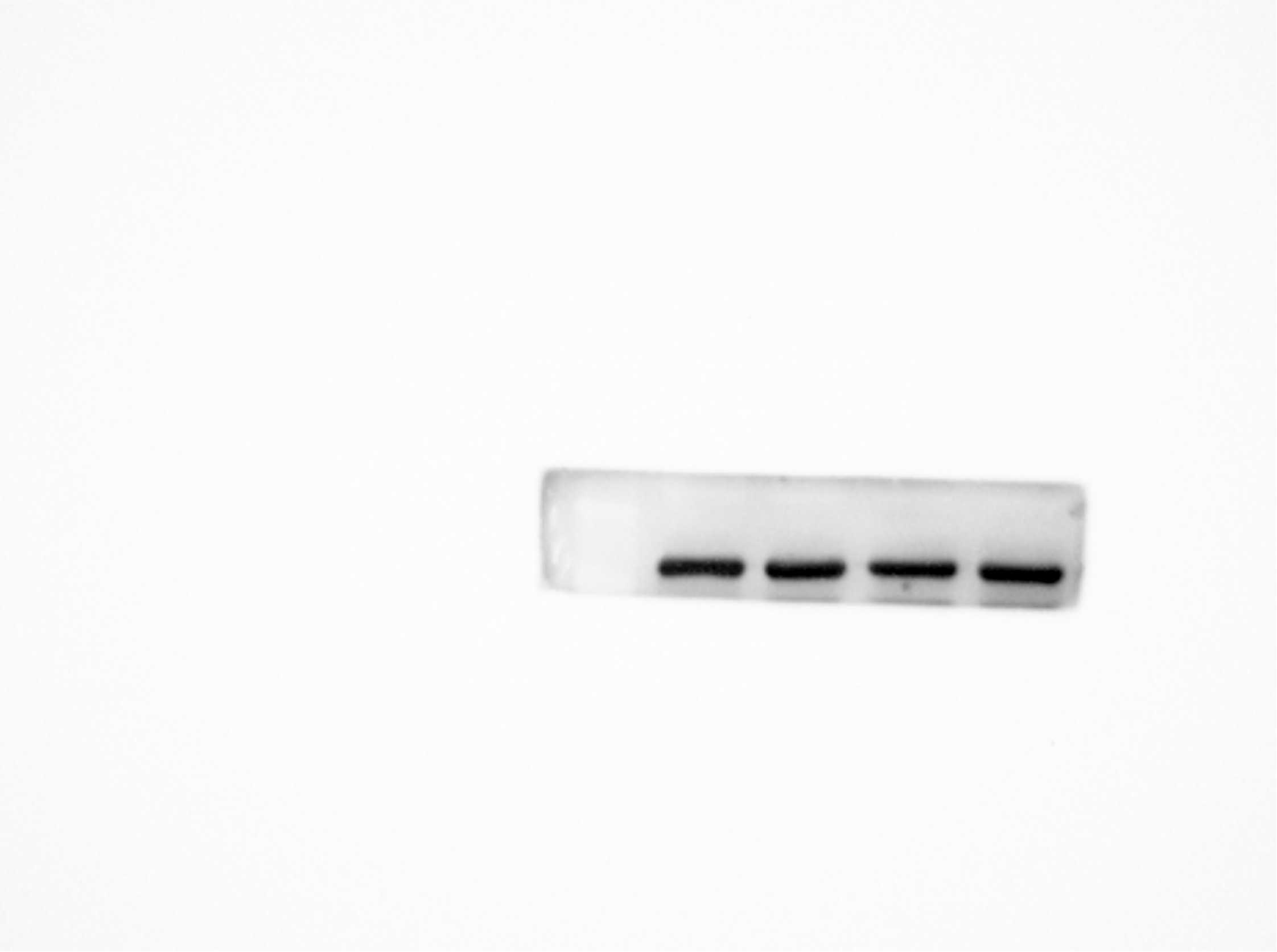

Supplement: Supplementary file 1 [file DataSheet1.ZIP › original data(supplement)/GAPDH-2.tif]

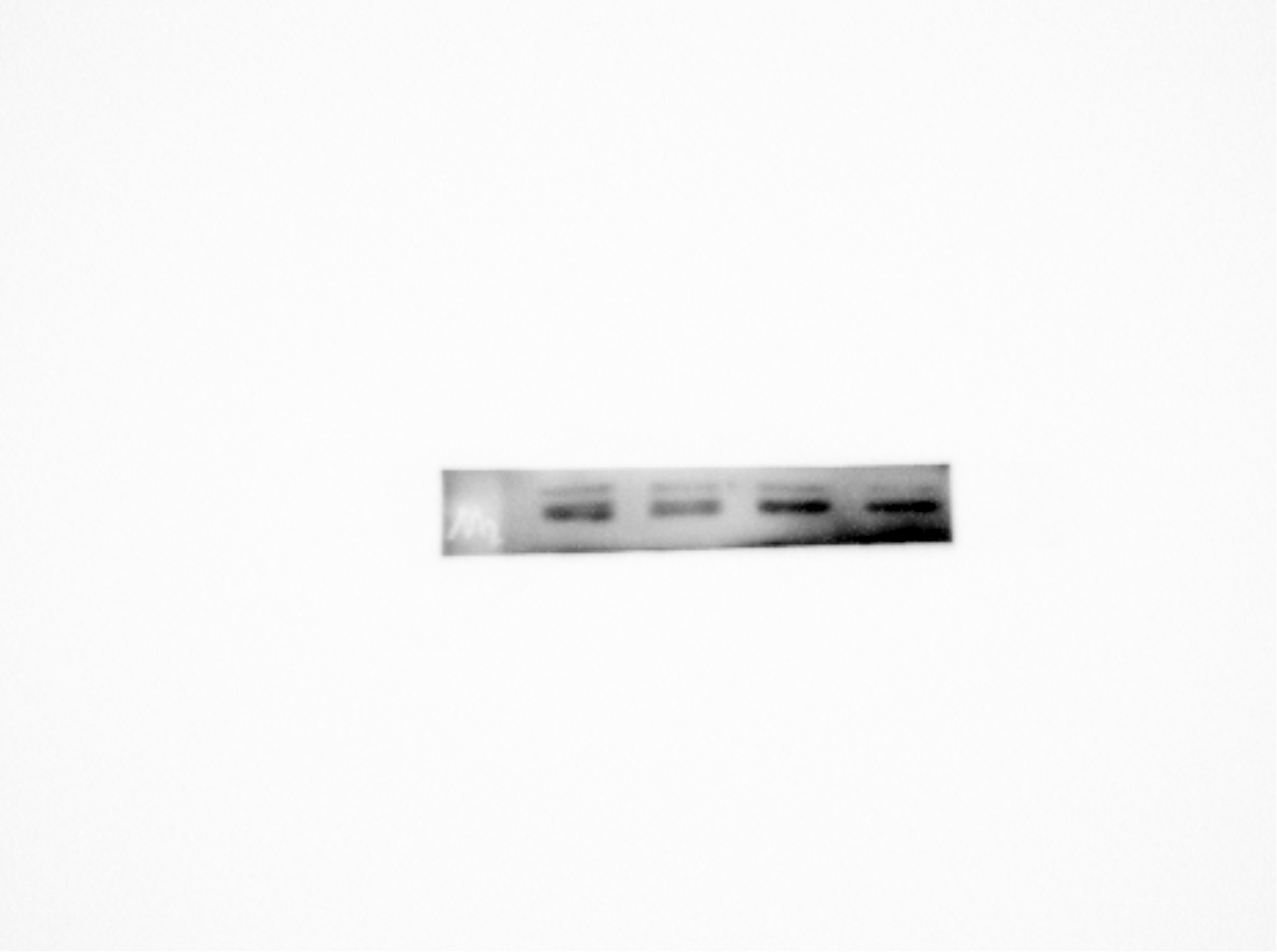

Supplement: Supplementary file 1 [file DataSheet1.ZIP › original data(supplement)/MARCH1.tif]

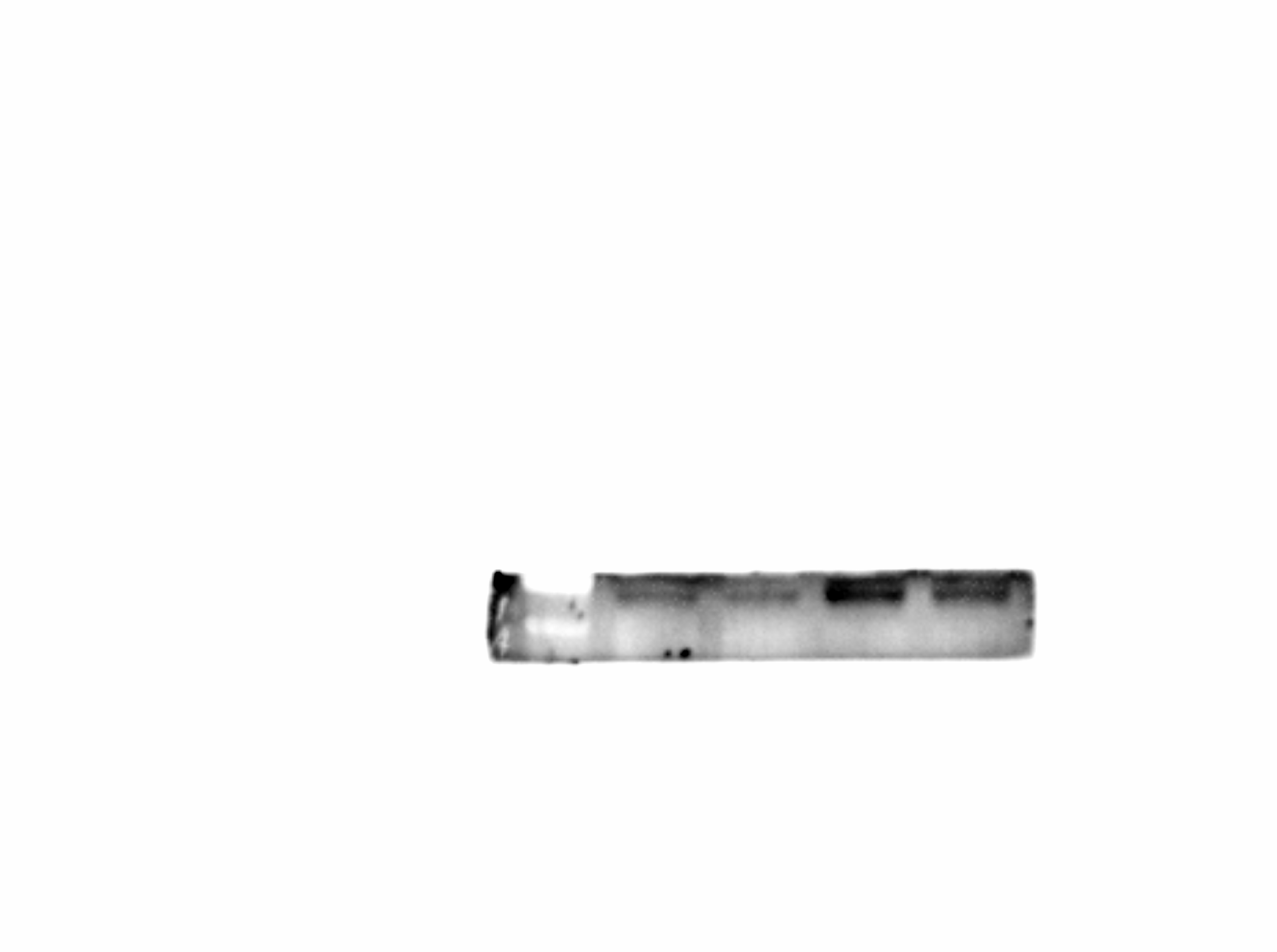

Supplement: Supplementary file 1 [file DataSheet1.ZIP › original data(supplement)/p-AMPK.tif]

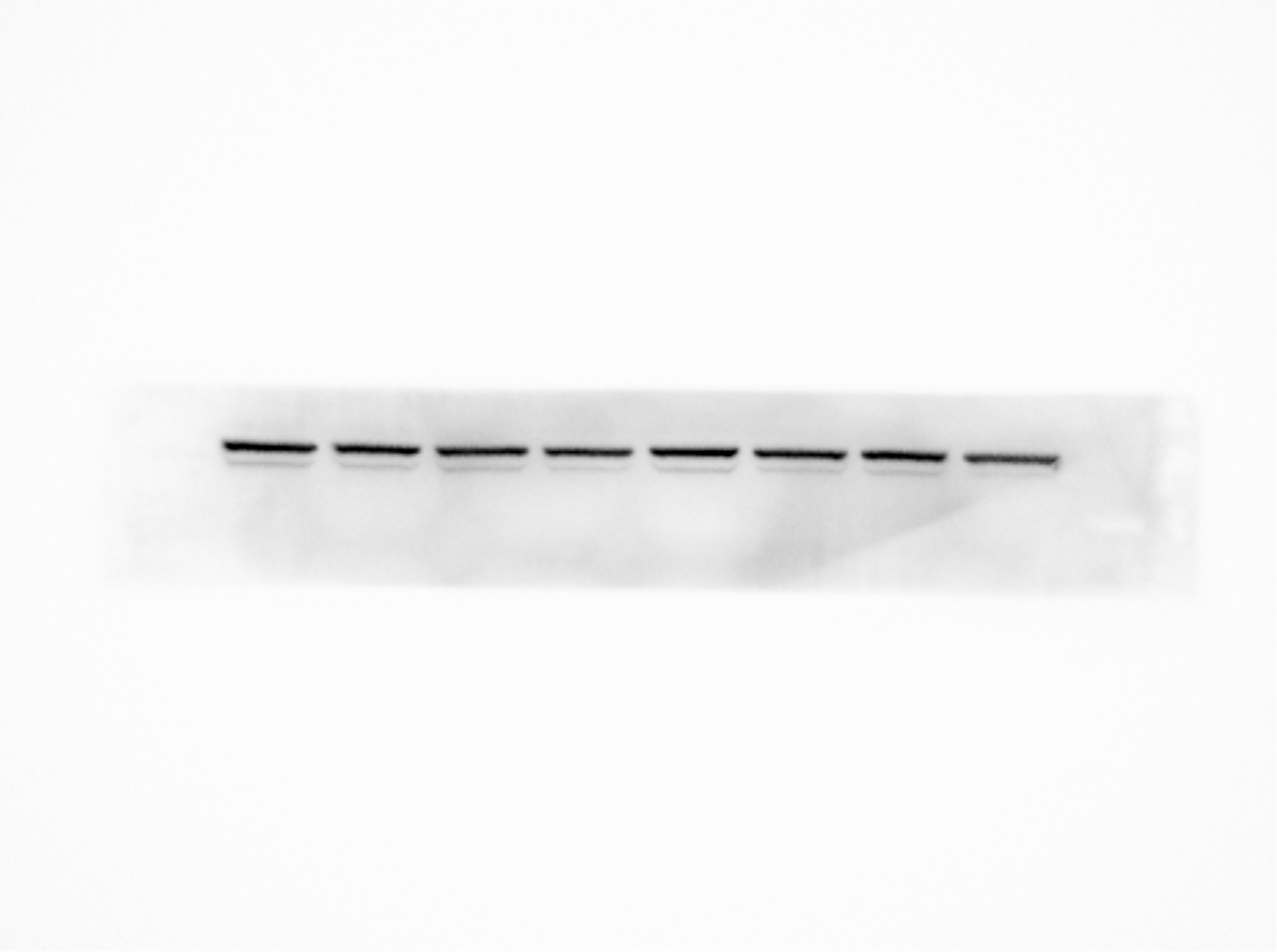

Supplement: Supplementary file 1 [file DataSheet1.ZIP › original data(supplement)/p-stat3.tif]
